# Potential Effects of Bee Products Against Hantavirus Infection: Potential Mechanisms of Action and Future Directions

**DOI:** 10.3390/life16060995

**Published:** 2026-06-12

**Authors:** Saad N. Al-Kahtani, Ahmed A. Rawwash, Amal Semmar, Sahar Gaber, Nabil M. Elwakeil, El-Kazafy A. Taha

**Affiliations:** 1 Plant Protection Science Program, Department of Arid Land Agriculture, College of Agricultural and Food Sciences, King Faisal University, Al-Ahsa 31982, Saudi Arabia; nelwakeil@kfu.edu.sa; 2 Department of Botany and Microbiology, College of Science, Al-Azhar University, Assiut Branch, Assiut 71524, Egypt; 3 Biotechnology, Environment and Health Laboratory, Saad Dahleb University-Blida 1, Blida 09000, Algeria; semmaramal13@gmail.com; 4 Honey Bee Research Department, Plant Protection Research Institute, Agricultural Research Center, Dokki, Giza 12611, Egypt; sahar.gaber85@gmail.com; 5 Department of Economic Entomology, College of Agriculture, Kafrelsheik University, Kafrelsheik 33516, Egypt; elkazafi.taha@agr.kfs.edu.eg

**Keywords:** Hantavirus, bee pollen, propolis, honey, bee venom, royal jelly, antiviral

## Abstract

Hantaviruses (HTVs) are lethal zoonotic pathogens responsible for hemorrhagic fever with renal syndrome and HTV cardiopulmonary syndrome; however, no specific antiviral treatments or vaccines have been approved. Bee products, such as propolis, honey, royal jelly, bee venom, and bee pollen, demonstrate extensive antiviral, anti-inflammatory, antioxidant, and immunomodulatory properties against various RNA and DNA viruses. No published research has directly evaluated bee products in relation to HTV infection. This review proposes a hypothesis-driven mechanistic framework suggesting that bioactive compounds from bee products may concurrently inhibit HTV replication, alleviate the cytokine storm, diminish oxidative stress, and maintain endothelial barrier integrity. We explicitly recognize the lack of direct experimental evidence regarding bee products’ efficacy against HTVs. Considering the mechanistic similarities with other enveloped viral infections and the recognized functions of NF-κB, Nrf2, and endothelial signaling pathways in HTV pathogenesis, we present a scientifically substantiated rationale for forthcoming research endeavors. The diverse bioactive compounds present in bee products including bee pollen, bee venom, honey, propolis, and royal jelly could provide a multifaceted strategy for inhibiting HTV pathology. We propose systematic in vitro, in silico, and in vivo investigations to assess the potential of bee-derived flavonoids, peptides, and fatty acids as adjunctive therapeutic strategies for HTV disease.

## 1. Introduction

Hantaviruses, belonging to the Hantaviridae family, are enveloped, negative-sense single-stranded RNA viruses that are naturally harbored by rodents and transmitted to humans mainly via the inhalation of aerosolized excreta [1,2]. Human infection results in two distinct clinical syndromes: hemorrhagic fever with renal syndrome (HFRS), caused by Old World HTVs like Hantaan and Seoul viruses, and HTV cardiopulmonary syndrome (HCPS), caused by New World viruses such as Sin Nombre and Andes viruses [3,4,5]. Hantaviruses rank among the deadliest zoonotic viral pathogens, infecting roughly 200,000 individuals globally each year, with a mortality rate of 35–40% [1]. Recent outbreaks, including a 2026 Andes virus cluster on an international cruise ship, highlight the persistent public health threat and the urgent necessity for effective therapeutic strategies [6].

The pathology of HTV disease is complex and multifaceted, with endothelial barrier dysfunction resulting from a dysregulated immune response, which includes cytokine storm, complement activation, and neutrophil/platelet activation [3]. The severity of the disease is significantly correlated with the elevated levels of pro-inflammatory cytokines (IL-6, TNF-α, IFN-γ). Vascular permeability is further exacerbated by coagulation irregularities (DIC, bradykinin release) and oxidative stress [5]. Supportive care is the primary treatment for the majority of HTVs, as there are no approved antivirals or vaccines. As a result, there is an immediate need for innovative adjunct therapies that address the fundamental inflammation and endothelial injury [7,8].

Bee products, including honey, propolis, royal jelly, bee venom, and bee pollen, have been employed in traditional medicine for centuries and are now being acknowledged for their diverse pharmacological properties [9]. Their extensive antiviral efficacy against both RNA and DNA viruses, including influenza, coronaviruses, herpes simplex virus (HSV), and HIV, has been demonstrated in recent studies [10,11]. These products exhibit substantial anti-inflammatory, antioxidant, and immunomodulatory properties in addition to their direct antiviral effects. They frequently function by modulating critical signaling pathways such as NF-κB and Nrf2 [12,13,14,15]. The most recent evidence suggests that specific compounds present in bee products can protect endothelial function and prevent pathological angiogenesis, which are directly related to HTV-induced vascular leakage [16].

This review establishes a hypothesis-driven mechanistic framework that examines the potential of bee products to disrupt the primary pathogenic pathways of HTV infection through their multi-target bioactivities. We explicitly acknowledge the absence of direct experimental evidence and offer a scientifically sound rationale for future research. The primary hypothesis posits that bioactive compounds derived from bee products can simultaneously inhibit viral replication, mitigate the cytokine storm, diminish oxidative stress, and preserve endothelial barrier integrity, thereby alleviating the severe pulmonary and renal complications associated with HTV disease.

## 2. Hantavirus Pathogenesis

**Figure 1:**
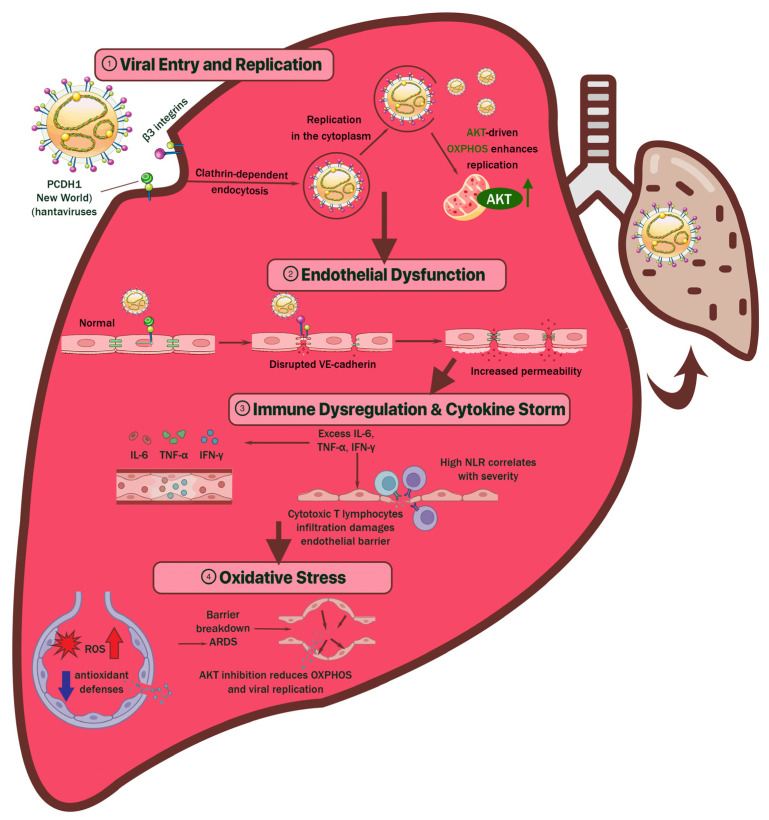
The pathogenesis of HTV.

The pathogenesis of HTV is a complex process that leads to severe clinical manifestations, including HTV Cardiopulmonary Syndrome (HCPS) and HFRS [17]. Zoonotic diseases induced by rodent-borne HTVs are characterized by viral entry and replication, significant endothelial dysfunction, severe immune dysregulation leading to a cytokine storm, and lung injury driven by oxidative stress [18] (Figure 1).

The initial phase involves viral entry and replication. Hantaviruses are enveloped, negative-sense RNA viruses that primarily target human endothelial cells, particularly in the lungs for those associated with HCPS and in the kidneys for those linked to HFRS [19,20]. Preliminary research suggested that β3 integrins (e.g., αVβ3) serve as principal entry receptors; however, later genetic knockout studies identified protocadherin-1 (PCDH1) as a crucial host factor for New World HTVs, such as Andes virus (ANDV) and Sin Nombre virus (SNV) [21,22]. These viruses bind to specific receptors on the cell surface, facilitating entry, usually through clathrin-mediated endocytosis. Following entry, the viral ribonucleoprotein complex is released into the cytoplasm, where replication occurs in perinuclear Golgi-associated factories [18]. It is crucial to acknowledge that HTVs are generally considered non-cytopathic; they do not directly lyse or destroy the infected endothelial cells. The non-cytopathic feature signifies that the notable pathology observed arises not from direct viral damage but from host responses and viral manipulation of cellular functions [23]. The replication of Hantaan virus (HTNV) is enhanced through mitochondrial oxidative phosphorylation (OXPHOS) via AKT activation [24]. The viral RNA-dependent RNA polymerase (RdRp) is essential for the transcription and replication of viral RNA, rendering it a prospective therapeutic target [25].

Endothelial dysfunction is a characteristic hallmark of HTV diseases and is crucial to the increased vascular permeability noted in both HCPS and HFRS [19]. The virus does not directly destroy endothelial cells; instead, it impairs their normal barrier functions. Pathogenic HTVs can bind to β3 integrins on endothelial cell membranes, and this interaction, even if not always mediating primary viral entry, is critical as it can activate intracellular signaling pathways that contribute to endothelial dysfunction and increased vascular permeability. This may lead to the phosphorylation and internalization of Vascular Endothelial cadherin (VE-cadherin); although both PCDH1 and VE-cadherin belong to the cadherin superfamily and mediate calcium-dependent cell–cell adhesion, they are distinct proteins with different structures, tissue distributions, and biological functions. Disruption of VE-cadherin-mediated junctions directly contributes to increased vascular permeability. In HCPS, this dysfunction manifests as high-permeability pulmonary oedema, characterized by capillary leakage, leading to plasma infiltration into the lung alveoli and subsequent acute respiratory distress syndrome (ARDS) [23]. The disruption of endothelial barriers can lead to substantial clinical consequences, including acute kidney injury in HFRS and cardiogenic shock in severe HCPS cases [26,27]. Endothelial dysfunction is a notable feature of various severe viral infections, including COVID-19, as it exacerbates microvascular health issues and is amplified by oxidative stress and inflammation [28].

Immune dysregulation and cytokine storm are critical components of HTV pathogenesis. In humans, HTV infection often elicits an exaggerated innate immune response, which substantially influences severe clinical outcomes [18]. Infected cells, particularly endothelial cells and macrophages, overproduce pro-inflammatory cytokines such as IL-6, TNF-α, and IFN-γ [2]. This unregulated systemic inflammatory response is known as a cytokine storm and is closely associated with disease severity and mortality [2,29]. In HCPS, the intensity of the cellular immune response, particularly the infiltration of the lungs by virus-specific cytotoxic T lymphocytes (CTLs), correlates with disease severity. The CTLs, while attempting to eradicate infected endothelial cells, inadvertently undermine the vascular barrier, leading to vascular leakage. The HTNV induces distinct macrophage responses that affect infection outcomes in humans relative to rodents, with the activation of inflammatory macrophages contributing to cytokine storm syndrome in humans [2]. The cytokine storm phenomenon is also observed in other severe viral infections, including COVID-19, where it contributes to acute respiratory distress syndrome (ARDS) and multi-organ dysfunction [29,30]. The elevated neutrophil-to-lymphocyte ratio (NLR) observed in acute HTV infection correlates with markers of disease severity, indicating systemic inflammation [27].

Oxidative stress and pulmonary injury substantially exacerbate HTV disease. The significant inflammatory response and endothelial dysfunction lead to an imbalance between reactive oxygen species (ROS) production and antioxidant defenses, resulting in oxidative stress. This redox imbalance intensifies the progression of lung injury and ARDS [29]. In HCPS, substantial fluid accumulation in the lungs, resulting from immune-mediated damage to the vascular barrier, causes pulmonary oedema and hypoxaemia. The damage results not only from direct viral action but is significantly intensified by oxidative stress, which can induce pyroptotic cell death and further compromise the alveolar-capillary barrier. Research has shown that inhibiting AKT activation can prevent the increase in OXPHOS levels caused by HTNV infection, thereby impeding viral replication [24]. The inflammatory processes and oxidative stress linked to HTV infections are analogous to those in other severe conditions, such as COVID-19, where endothelial dysfunction and inflammation are crucial in the onset of ARDS [31].

## 3. Composition and Bioactive Compounds of Bee Products

The complex chemical compositions of bee products, which are contingent upon their botanical origin, geographic location, bee subspecies, harvest season and processing methods, are responsible for their pharmacological activities [32,33,34]. Each product is composed of a distinctive array of bioactive compounds that exhibit complementary and overlapping mechanisms of action.

Honey is a multifaceted substance that comprises approximately 80% sugars, as well as water, enzymes (glucose oxidase, catalase), amino acids, vitamins, mineral elements, and polyphenolic compounds [33,35]. Phenolic acids (e.g., caffeic acid, ferulic acid, p-coumaric acid), flavonoids (e.g., quercetin, kaempferol, luteolin, apigenin, galangin), and methylglyoxal (MGO), a significant antimicrobial component of Manuka honey, are among the key bioactive constituents. Honey’s antiviral, anti-inflammatory, and antioxidant properties are primarily due to its polyphenolic content and the generation of hydrogen peroxide through glucose oxidase activity [36,37].

Propolis is a resinous substance that bees collect from tree buds and plant exudates, and it is combined with bee secretions and wax. Its composition is highly variable, but it is generally rich in polyphenols, including flavonoids (chrysin, quercetin, galangin, kaempferol, apigenin, pinocembrin), phenolic acids (caffeic acid, p-coumaric acid, ferulic acid), and their esters, specifically caffeic acid phenethyl ester (CAPE) and artepillin C. The CAPE has become a compound of particular significance due to its potent antiviral and anti-inflammatory properties [16,38].

Royal jelly is a creamy secretion that is exclusively consumed by queen bees and young larvae. It is produced by the hypopharyngeal and mandibular glands of nurse worker bees. Water (60–70%), proteins (12–15%), sugars (10–16%), lipids (3–8%), and minor constituents such as vitamins, mineral elements, and polyphenols comprise its bioactive components [39]. 10-hydroxy-2-decenoic acid (10-HDA) is the distinctive fatty acid with immunomodulatory and anti-inflammatory properties, and major royal jelly proteins (MRJPs) are the primary bioactive molecules. These proteins consist of nine distinct water-soluble proteins. The primary antiviral and immunomodulatory effectors in royal jelly have been identified as MRJPs [40,41].

Bee venom is a complex mixture of bioactive amines, enzymes, and peptides, with a minimum of 18 pharmacologically active components. The primary component, melittin, is a 26-amino-acid amphipathic peptide that disrupts lipid membranes and demonstrates extensive antimicrobial and antiviral properties, accounting for 40–60% of dry weight. Other significant constituents that contribute to its biological effects include apamin, adolapin, mast cell degranulating peptide, and phospholipase A2 [42,43].

Bee pollen is a composite of pollen grains that have been agglutinated with nectar/honey and bee salivary secretions. It is exceedingly abundant in nutrients, such as proteins (10–40%), carbohydrates (30–60%), lipids (1–10%), vitamins, mineral elements, and a diverse selection of polyphenolic compounds, including flavonoids and phenolic acids [34,44,45,46]. The potent antioxidant, anti-inflammatory, and immunomodulatory properties of bee pollen are attributed to its flavonoid content, which includes quercetin, kaempferol, myricetin, rutin, and apigenin [47,48].

## 4. Antiviral Effects of Bee Products

The broad-spectrum antiviral capabilities of bee products against a variety of human and animal viruses have been demonstrated in numerous studies over the past decade. This provides a strong foundation for hypothesizing their potential impact on HTV. The effects of bee products on different types of viruses (RNA and DNA), showing the mechanism of action of each bee product on each virus, are displayed (Table 1).

## 5. Mechanistic Intersection Between Bee Products and HTV Pathogenesis

Bee products demonstrate a variety of biological activities that imply a potential role in the modulation of HTV infection pathways, including viral replication, immune dysregulation, oxidative stress, and endothelial dysfunction.

### 5.1. Antiviral Mechanisms Against HTV Replication

The antiviral compounds present in bee products may be effective against HTVs, which are enveloped RNA viruses [3]. Research has demonstrated that propolis flavonoids, including galangin and chrysin, can inhibit viral enzymes such as SARS-CoV-2 3CLpro and RdRp [61]. This implies that comparable compounds may disrupt the replication and assembly of HTV RNA-dependent RNA polymerase or other critical viral proteins [51]. Melittin, a primary peptide in bee venom, disrupts viral envelopes by forming pores in lipid bilayers, which could directly neutralize circulating HTV particles, preventing their entry into host cells or rendering them non-infectious [59]. Honey’s virucidal activity, which is partially attributed to the production of hydrogen peroxide and methylglyoxal, may also have direct antiviral effects on the viability of HTVs [49].

### 5.2. Anti-Inflammatory and Immunomodulatory Effects

A cytokine storm is a term that frequently describes the exaggerated host inflammatory response that is the primary driver of HTV pathogenesis [2]. Bee products are acknowledged for their immunomodulatory and anti-inflammatory capabilities [9]. Propolis, which is abundant in CAPE, inhibits the activation of nuclear factor-kappa B (NF-κB), a master regulator of inflammation. Propolis has the potential to reduce the excessive production of pro-inflammatory cytokines, including TNF-α, IL-6, and IL-1β, as well as chemokines that are involved in HTV-induced cytokine storms, by suppressing NF-κB [11]. Royal jelly contains 10-hydroxy-2-decenoic acid (10-HDA) and 10-hydroxydecanoic acid (10-HDA), which have been shown to suppress NF-κB activation and reduce IL-6 levels in LPS-induced inflammatory models. This could translate to a reduction in the severity of immune dysregulation observed in HTV infections [62]. By regulating signaling pathways that are involved in the metabolism of arachidonic acid and the degranulation of mast cells, bee venom components, such as melittin, apamin, and phospholipase A2, exhibit substantial anti-inflammatory effects [63]. In addition, honey and bee pollen contain polyphenols that can reduce inflammatory responses by inhibiting inflammatory mediators and modulating immune cell activity [64].

### 5.3. Antioxidant Properties and Oxidative Stress Reduction

Endothelial damage and overall pathology are exacerbated by HTV infection, which results in substantial oxidative stress [2]. Flavonoids, phenolic acids, and enzymes are all excellent sources of natural antioxidants, including bee products [65]. These compounds have the ability to protect cells from oxidative damage, enhance endogenous antioxidant enzyme systems, such as the NRF2/HO-1 pathway, and scavenge free radicals. For example, the NRF2/HO-1 axis is activated by propolis flavonoids [61]. Bee products could theoretically preserve endothelial integrity and reduce tissue injury during HTV infection by reducing oxidative stress [65].

### 5.4. Endothelial Protective Effects

In HTV disease, the endothelial barrier rupture is a critical event that results in severe vascular leakage. The antioxidant and anti-inflammatory properties of bee products are directly relevant to the protection of endothelial cells [13]. Propolis and bee pollen contain polyphenols that can fortify endothelial junctions, mitigate vascular permeability, and safeguard endothelial cells from oxidative and inflammatory insults [61,65]. For example, the activation of the NRF2/HO-1 pathway by bee pollen polyphenols has the potential to reduce oxidative endothelial injury [61]. Hypothetically, the components of royal jelly could stabilize endothelial function by mitigating inflammation-induced damage [62].

**Figure 2:**
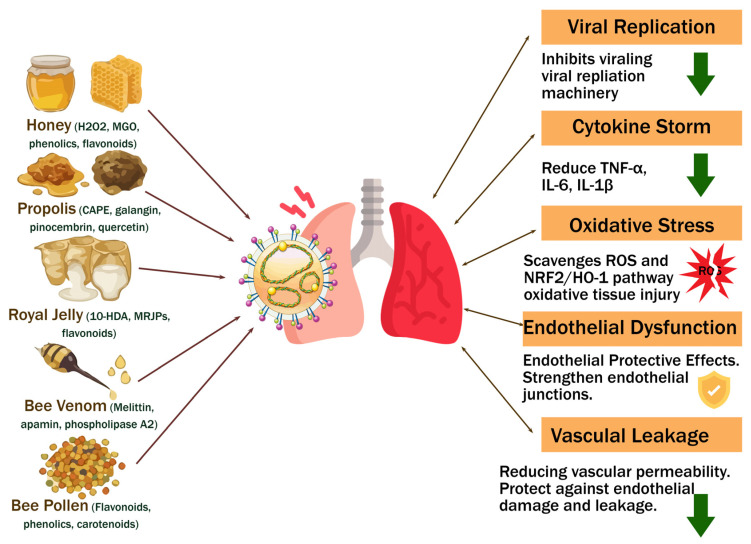
Hypothetical mechanism of bee products against HTV infection.

Multiple pathways that are implicated in HTV disease are targeted by bioactive constituents of bee products. As an overarching hypothesis, bee-derived flavonoids, peptides, and acids may interact with HTV proteins and host signaling to reduce viral load, oxidative injury, cytokine dysregulation, and endothelial dysfunction (Figure 2). Despite the absence of direct evidence, this mechanism-based rationale implies a potential supportive role that warrants further investigation.

## 6. Research Gap and Scientific Hypothesis

It is important to note that, despite the mechanistic similarities, no published research has directly evaluated bee products in relation to HTV infection. A recent review of bee product antivirals examined 51 studies (1992–2023) on bee-derived antivirals but did not identify any that addressed HTVs [10]. This discrepancy is substantial due to the fact that HTVs are incurable, whereas phylogenetically related viruses, including influenza and arenaviruses, have been suppressed by compounds derived from bees. The biosafety requirements of HTVs and their unique outbreak characteristics may be the reason for the absence of research. However, focused investigations are necessary in light of the strong preclinical evidence supporting the efficacy of bee bioactive compounds against enveloped viruses and inflammatory pathways.

Scientific Hypothesis: We contend that bioactive compounds found in bee products have the potential to disrupt critical pathogenic pathways in HTV infection, thereby reducing viral replication, oxidative stress, cytokine-mediated endothelial activation, and inflammatory tissue damage. This mechanistic hypothesis is based on the following: (1) the established antiviral and immunomodulatory properties of bee constituents in various infections [11,41], (2) the recognized functions of these pathways in HTV pathology [5], and (3) initial docking/in silico analyses that indicate that bee flavonoids interact with viral proteins in other coronaviruses, including SARS-CoV-2. For example, research has demonstrated that propolis flavonoids, including chrysin and galangin, as well as honey flavonoids like quercetin, can effectively bind to critical viral enzymes, including the SARS-CoV-2 main protease and RNA-dependent RNA polymerase, indicating their potential to inhibit viral replication [49,61,65]. This suggests that a similar strategy in the context of HTV glycoproteins or polymerase may produce advantageous outcomes. The hypothesis deliberately refrains from asserting any established therapeutic efficacy; it positions bee products as potential subjects for further investigation, in accordance with an emerging treatment paradigm.

## 7. Future Perspectives and Research Recommendations

Future research should systematically investigate the potential of bee products as supportive agents against HTV infection, transitioning from in silico predictions to rigorous experimental validation.

In Vitro Studies: Assess the antiviral properties of honey, royal jelly, and bee venom compounds as well as bee pollen and propolis extracts in cell culture. Utilize human endothelial or monocyte cell lines that have been infected with HTVs. Assess viral replication in the presence of bee compounds (standardized extracts or purified flavonoids, melittin) using plaque assays or RT-qPCR. In the presence or absence of treatment, evaluate endothelial permeability (trans-endothelial resistance, VE-cadherin expression) and cytokine production (IL-6, TNF). Evaluate the potential for synergy in combinations.

Mechanistic Assays and Molecular Docking: Conduct in silico docking of critical bee-derived molecules (CAPE, chrysin, quercetin, melittin fragments, MRJP-derived peptides) to HTV targets, including viral glycoprotein Gn/Gc (entry mediators) [66], RdRp or host receptors (β3 integrin). If promising binders are identified, these should be verified through enzymatic assays, such as RdRp inhibition. Examine the impact of signaling on host cells, such as whether CAPE inhibits the activation of NF-κB in infected cells that has been induced by HTV.

The utilization of standardized bee products: In order to guarantee the reproducibility, reliability, and clinical relevance of research on bee products, it is mandatory to utilize chemically well-characterized and standardized preparations. Standardization necessitates the implementation of stringent quality control measures to guarantee consistent concentrations of critical bioactive markers. For example, propolis extracts should be standardized to a specific flavonoid content (e.g., chrysin, galangin, CAPE), whereas royal jelly could be standardized based on 10-HDA or MRJP content. This characterization is essential for the evaluation of antiviral potency against HTVs in in vitro and in vivo studies, as well as for future clinical translation.

In Vivo Models: Test the efficacy of bee products by utilizing established animal models of HTV (e.g., Syrian hamsters with Andes virus or non-human primates with Puumala). Standardized propolis extract, royal jelly, or bee venom melittin should be administered prophylactically or therapeutically at non-toxic doses. Assess survival, cytokine levels, lung/kidney pathology, and viral loads. Additionally, evaluate biomarkers of endothelial function, such as the vascular leak index. Mechanistic endpoints (cytokine profiles, oxidative markers) should be prioritized in outcomes over cure claims.

Formulation and Delivery: In light of the occasionally low bioavailability of bee compounds, it is recommended to investigate the use of formulations (e.g., nanoparticles, liposomes) to improve the delivery of the compound to the endothelium or lungs. For instance, nano-encapsulated flavonoids or melittin may enhance activity at infection sites while simultaneously decreasing systemic toxicity.

Strategies that work in conjunction: Examine the use of bee products in conjunction with other treatments. Could propolis complement passive antibody therapy or enhance interferon responses to HTV? Is it possible for honey or royal jelly to alleviate HTV-associated coagulopathy by means of antioxidant effects?

Preclinical Safety: Evaluate the potential adverse effects in the context of HTV disease. It is important to note that allergic reactions can be induced by bee venom, and high doses of antioxidants can paradoxically affect immune signaling. Efficacy tests should be accompanied by toxicology studies.

The identified gap could be addressed by any of these research directions. It is a valuable scientific inquiry to determine whether accessible natural products, such as bee derivatives, could mitigate disease, given the global distribution of HTVs and climate-related emergence. It would be advantageous for researchers in virology and apitherapy to collaborate.

## 8. Conclusions

Characterized by severe clinical outcomes and a critical absence of approved antiviral therapies, HTV infections continue to present a formidable challenge to global health. Innovative therapeutic strategies are required due to the complex pathogenesis, which is characterized by viral manipulation of host responses, endothelial barrier disruption, unchecked immune activation, and oxidative stress. A mechanistic hypothesis has been systematically delineated in this review, which posits that the diverse bioactive compounds present in bee products such as honey, propolis, royal jelly, bee venom, and bee pollen could provide a multifaceted strategy for inhibiting HTV pathology. Their potent anti-inflammatory, antioxidant, and immunomodulatory capabilities, in conjunction with their well-documented antiviral effects against a spectrum of RNA and DNA viruses, are directly aligned with the key pathogenic pathways of HTV disease. In particular, these compounds are believed to disrupt viral replication, mitigate the deleterious cytokine storm, mitigate oxidative damage, and re-establish endothelial integrity. Despite the absence of direct experimental validation against HTVs, the established biological activities of bee products and the compelling evidence from other viral infections provide a robust foundation. In order to empirically test this hypothesis, elucidate precise molecular mechanisms, and ultimately translate these promising natural interventions into effective adjunct therapies for HTV infection, future research must prioritize rigorous in vitro and in vivo studies. This will address a critical unmet medical need. 

## Figures and Tables

**Table 1:** Effect of bee products on a variety of viruses.

Bee Product	Virus Type (RNA/DNA)	Mechanism of Action	Methods Used (In Vitro/In Vivo)	Result	Ref.
Honey	RNA (SARS-CoV-2 models)	Direct virucidal effects (osmotic/H_2_O_2_ and phytochemicals); inhibition of viral entry; immunomodulation (↑ IFN, ISGs).	in vitro; some in vivo/animal and mechanistic studies	Reduced viral infectivity and replication in cell models; enhanced innate antiviral responses in animal/cell studies.	[49]
	DNA (HSV-1)	Inhibition of viral binding and entry, suppression of ROS-NLRP3 inflammasome pathway, and reduction in pro-inflammatory cytokines.	in vitro (cell culture)	Korean chestnut honey (KCH) inhibited HSV-1 infection through virucidal effects and by blocking viral replication.	[50]
Propolis	RNA and DNA (influenza, SARS-CoV-2 models; HSV DNA)	Polyphenols/flavonoids inhibit attachment and replication; anti-inflammatory and immunomodulatory effects.	in vitro; some in vivo and small clinical trials	Broad-spectrum inhibition of viral infectivity/replication in vitro; some clinical signals of symptom reduction in small trials.	[51]
	RNA Human coronavirus OC43 (HCoV-OC-43)	Interference with viral adsorption, direct virucidal effect, and inhibition of viral replication during the intracellular cycle.	in vitro (CPE inhibition test, plaque reduction)	Bulgarian propolis extracts showed strong activity against HCoV-OC-43 replication; the effect was more pronounced against enveloped viruses.	[52]
Royal jelly	RNA (SARS-CoV-2)	Major royal jelly protein fraction (PF_50_) inhibits viral entry (spike-ACE2 interaction) and suppresses RdRp activity.	in vitro (lentiviral pseudotype system)	PF_50_ efficiently blocked entry of SARS-CoV-2 variants (IC_50_ = 7.25 µM for UK variant) and inhibited RdRp activity (IC_50_ = 29.93 µM).	[53]
	RNA hepatitis C virus (HCV), human immunodeficiency virus (HIV)/DNA hepatitis B virus (HBV)	Major royal jelly proteins (MRJPs) target viral enzymes such as RNA-dependent RNA polymerase and reverse transcriptase.	in vitro	MRJPs demonstrated antiviral effects against HCV, HBV, HIV, and SARS-CoV-2 by interfering with specific stages of the viral life cycle.	[54]
Bee venom	DNA (HSV-1, HSV-2)	Direct inactivation of viral particles, disruption of lipid membranes, and interference with viral attachment to host cells.	in vitro (plaque reduction, virucidal assays)	Melittin from *Apis mellifera* and *A. florea* showed EC_50_ values of 4.90 µg/mL (HSV-1) and 4.39 µg/mL (HSV-2); melittin at 5 µg/mL produced the highest direct virucidal effect.	[55]
	RNA (influenza, RSV, HIV)	Melittin and phospholipase A_2_ (PLA_2_) disrupt viral envelopes, inhibit viral attachment, and modulate host signalling pathways (e.g., NF-κB, JAK/STAT3).	in vitro	Bee venom demonstrates broad-spectrum activity against enveloped RNA viruses (influenza, HIV, RSV) and non-enveloped viruses.	[56]
Bee pollen	RNA (influenza A H1N1)	Likely mediated by proteinaceous compounds and specialised metabolites; the exact mechanism is still under investigation.	in vitro (real-time PCR)	Bee-collected pollen and bee bread showed significant anti-IAV activity with IC_50_ values ranging from 0.022 to 10.04 mg/mL and SI values up to 338.64.	[57]
Honey + Royal jelly	RNA (SARS-CoV-2 targets studied)	Protease/enzyme binding (bioactive flavonoids, fatty acids); immune modulation.	in silico docking; limited in vitro follow-up	Docking identified royal jelly/honey compounds with predicted binding to SARS-CoV-2 main protease; suggests candidates for in vitro testing.	[58]
Honey + Propolis	RNA (e.g., SARS-CoV-2)	Flavonoids (e.g., rutin, naringin) inhibit viral-spike fusion with host cells and viral replication; propolis also blocks viral entry and boosts immune response.	in vitro (Vero E6 cells); In vivo (hospitalized COVID-19 patients receiving combination of green propolis or honey + *Nigella sativa*)	Both honey and propolis inhibit SARS-CoV-2 infection in cell culture; clinical data show earlier viral clearance, symptom recovery and reduced mortality when used as adjuvant treatment.	[38]
Honey Bee Venom + Propolis Extract	RNA (Deformed wing virus, Black queen cell virus, *Varroa destructor* virus-1, Kakugo virus)	Direct virucidal activity; reduces viral RNA levels in infected cell cultures; mechanism may involve disruption of viral envelopes and interference with replication.	in vitro (honey bee cell lines)	HBV and propolis extract exhibited a significant reduction in all tested viruses; low concentrations were non-toxic and effective, suggesting potential as natural antiviral supplements for honeybees.	[59]
Royal Jelly + Propolis + Bee Pollen	RNA (SARS-CoV-2)	Anti-inflammatory, antioxidant and immunomodulatory actions; alleviates symptoms and reduces disease duration; likely interferes with viral propagation without direct virucidal effect.	in vivo (randomized, open-label, controlled trial in 50 COVID-19 patients)	Supplementation with the three products significantly reduced total symptom duration and time to return to work compared to standard care alone. Functional class improved more in the intervention group.	[60]

## Data Availability

All data generated or analyzed during this study are included in this published article.
